# *KLK10* exon 3 unmethylated PCR product concentration: a new potential early diagnostic marker in ovarian cancer? - A pilot study

**DOI:** 10.1186/s13048-018-0407-y

**Published:** 2018-04-24

**Authors:** Mustafa A. El Sherbini, Amal A. Mansour, Maha M. Sallam, Emtiaz A. Shaban, Zeinab A. Shehab ElDin, Amr H. El-Shalakany

**Affiliations:** 1 Medical Biochemistry Department, Faculty of Medicine, Ain Shams University, Cairo, Egypt; 2 Gynecologic Oncology Unit, Ain Shams University Maternity Hospital, Cairo, Egypt

**Keywords:** Ovarian cancer, CA125, KLK10, KLK6, *KLK10* exon 3 methylation

## Abstract

**Background:**

*KLK10* exon 3 hypermethylation correlated to tumor-specific lack of *KLK10* expression in cancer cell lines and primary tumors. In the present study we investigate the possible role of *KLK10* exon 3 methylation in ovarian tumor diagnosis and prognosis.

**Results:**

Qualitative methylation-specific PCR (MSP) results did not show statistically significant differences in patient group samples (normal and tumor) where all samples were positive only for the unmethylated-specific PCR except for two malignant samples that were either doubly positive (serous carcinoma) or doubly negative (Sertoli-Leydig cell tumor) for the two MSP tests. However, *KLK10* exon 3 unmethylated PCR product concentration (ng/μl) showed statistically significant differences in benign and malignant patient group samples; mean ± SD (n): tumor: 0.077 ± 0.035 (14) and 0.047 ± 0.021 (15), respectively, *p*-value = 0.011; and normal: 0.094 ± 0.039 (7) and 0.046 ± 0.027 (6), respectively, *p*-value = 0.031. Moreover, ROC curve analysis of *KLK10* exon 3 unmethylated PCR product concentration in overall patient group samples showed good diagnostic ability (AUC = 0.778; *p*-value = 0.002). Patient survival (living and died) showed statistically significant difference according to preoperative serum CA125 concentration (U/ml); median (n): 101.25 (10) and 1252 (5), respectively, *p*-value = 0.037, but not *KLK10* exon 3 unmethylated PCR product concentration (ng/μl) in overall malignant patient samples; mean ± SD (n): 0.042 ± 0.015 (14) and 0.055 ± 0.032 (7), *p*-value = 0.228.

**Conclusion:**

To the best of our knowledge, this is the first report on *KLK10* exon 3 unmethylated PCR product concentration as potential early epigenetic diagnostic marker in primary ovarian tumors. Taken into account the limitations in our study (small sample size and semi-quantitative PCR product analysis) further studies are strongly recommended.

## Background

DNA methylation is one of the well-studied epigenetic modifications in DNA/chromatin metabolism. It is a dynamic process and involves the reversible and heritable methylation of the 5′ carbon of cytosine residues to yield 5-methylcytosine (5-MC) [1]. The reaction belongs to one carbon metabolism where DNA methyltransferases (DNMT1, DNMT3a and DNMT3b) are the biocatalysts and S-adenosylmethionine (SAM) is the methyl-donor [2]. Besides having important physiological roles in cell differentiation, development and gene regulation [3], DNA methylation can provide clues to other physiological processes, e.g. cell and tissue aging [4] and establishment of memory [5].

In DNA the cytosine residues occur either in frequencies that are far less than expected or in CpG-rich short stretches (0.5–4 kbp) in gene promoters and other regulatory regions known collectively as CpG islands [6]. In the CpG context the two cytosines on the opposing DNA strands are usually symmetrical as for their methylation status, i.e. both are either unmethylated or methylated [7]. The effect of DNA methylation on gene regulation may differ according to the context in which it occurs; however, in CpG-rich gene promoters it is well known to share in gene silencing [8]. Deregulated gene methylation was implicated in several diseases including cancer [9]. Nonetheless, aberrant gene methylation in cancer can be a promising diagnostic and prognostic target in tumor and naked DNA samples; e.g. in lung cancer [10].

The kallikrein-related peptidase 10 gene (*KLK10*) is one of 15 members in a serine protease gene subfamily located in tandem on chromosome 19q13.3–13.4 [11]. The gene product (KLK10) is a secreted protein found in normal human mammary epithelial cells (MECs) but downregulated or absent in the majority of human breast cancer cell lines [12]. *KLK10* has a wide tissue expression [13] and is regulated by mechanisms that include steroid hormones [14] and micRNA [15]. The gene was reported as candidate tumor suppressor in some cancer types, e.g. in prostate cancer cells [16]; however, it showed contrasting expression profiles in different cancers, e.g. underexpressed in breast [17], testis [18] and prostate [19], and overexpressed in ovary [20], colon [21], and pancreas [22].

Liu and colleagues did not find mutation in *KLK10* gene in different cancer types [12]. Indeed, tumor-specific lack of *KLK10* expression correlated to *KLK10* exon 3 hypermethylation in a majority of cell lines and in primary breast cancer [23]. The rationale behind such correlation may be understood as *KLK10* exon 3 methylation was shown to perfectly follow that of *KLK10* gene promoter [24]. Results of previous works were consistent about the confinement of *KLK10* exon 3 hypermethylation in malignant samples; but not normal ones, in cell lines [23–25] and in primary tumors [23–26]. However, in some sample sets *KLK10* expression did not show simple correlation with *KLK10* exon 3 methylation [23–26]. In the present study, *KLK10* exon 3 methylation is assessed for its possible role in the biology, diagnosis and/or prognosis of ovarian tumors.

## Methods

### Patients and samples

This is a further study for our previous work on serum KLK6 and 10 in ovarian cancer patients [27]. The protocol of the present study was approved by the Ethics and Research Committee of Ain Shams University.

Patients’ demographic data and preoperative serum marker levels (CA125, KLK10 and KLK6) were taken from our previous data as mentioned above. Patients or their relatives were contacted from October 2012 to January 2013 to collect patient follow-up data and have their written informed consent.

The studied samples were archival FFPE-ovarian tissue samples that are available at the Gynecologic Oncology Unit, Ain Shams University Maternity Hospital. The included samples were tumors and their contralateral normal ovarian samples (for some cases), while non-neoplastic ovarian masses, i.e. inflammation and endometriosis, were excluded.

### Experimental protocol

Using histopathology microtome (microTec® *cut 4050*, UK) 10 to 15 tissue sections, each of ~ 2 × 2 cm in diameter and 1–2 μm thickness were cut and put in clean and autoclaved 1.5 ml microcentrifuge tube, one day before DNA extraction. An H&E stained tissue section was prepared and examined microscopically for each sample.

### DNA extraction

Commercially available QIAamp DNA FFPE Tissue Kit (Qiagen) was used. The steps of the experiment were done according to the product insert provided by the manufacturer. Xylol (Sigma-Aldrich, Germany) was used to dissolve the paraffin and 98–100% ethanol (Sigma-Aldrich, Germany) was used to remove the xylol and to reconstitute the buffers. The concentration of the extracted pure DNA was measured spectrophotometrically (Ultraspec® 1000, Amersham Pharmacia Biotech, Cambridge, England) at 260 nm and the DNA samples were stored at − 80 °C until the time of DNA sodium bisulfite treatment.

### Sodium bisulfite treatment of DNA

One μg of extracted DNA was treated by sodium bisulfite using commercially available EpiTect Bisulfite Kit (Qiagen). The steps of the experiment and the thermal cycler program were done according to the product insert given by the manufacturer for FFPE-samples. The sodium bisulfite converted DNA samples were kept at − 80 °C until the time of MSP experiment.

### MSP for *KLK10* exon 3

Commercially available HotStarTaq Master Mix kit (Qiagen) was used. Methylated- and unmethylated-specific PCRs were run in parallel in separate PCR tubes where each tube contained either methylated- or unmethylated-specific primer pair, respectively. The two primer pairs were provided by Invitrogen (USA), as described elsewhere [23]. Each PCR reaction contained ~ 0.5 μg sodium bisulfite-treated DNA sample. The concentrations of the PCR reaction components were made according to the manufacturer’s instructions in the product insert. A methylation positive control MSP was done using 2 μg (1 μg for each MSP test type) fully methylated DNA (EpiTect® Control DNA (human), methylated and bisulfite converted (100), Qiagen). The thermal cycler program was as follows: 1 cycle of 95 °C for 15 min; 35 cycles of 94 °C for 1 min, 55 °C for 1 min, and 72 °C for 1 min; and 1 cycle of 72 °C for 10 min.

### Capillary gel electrophoresis

PCR products were assessed by capillary gel electrophoresis using QIAxcel system (Qiagen). A QIAxcel kit (QIAxcel DNA High Resolution Kit (1200)) was used and the experiment steps were done according to the manufacturer’s instructions in the product insert. Determination of DNA fragment size and concentration was done using the BioCalculator software provided with the QIAxcel (Qiagen).

### Statistical analysis

Statistical analysis of data was done using SPSS (version 15.0 for Windows). *p* < 0.05 was considered the cutoff value for significance. Chi-square test (χ^2^) was used to test the association of categorical data. Parametric or non-parametric tests were used to compare mean ± SD (Student *t*-test or ANOVA test) or median and interquartile range (Mann-Whitney test or Kruskal Wallis test) in two or more populations, respectively, involving independent samples. Spearman’s test was used to evaluate correlations between continuous variables. A receiver operating characteristic (ROC) curve was used to illustrate the diagnostic properties of a test on a numerical scale. Marker’s diagnostic parameters were calculated as follows: sensitivity = true positive tests/ all positive samples by gold standard test; specificity = true negative tests/ all negative samples by gold standard test; positive predictive value (PPV) = true positive tests/ all positive tests; negative predictive value (NPV) = true negative tests/ all negative tests; and accuracy = true positive and true negative tests/all positive and all negative tests.

## Results

### Patients and samples

This study included 14 benign and 16 malignant ovarian tumor patients. Patient clinicopthological data are shown in Table 1. There were no statistically significant differences of patient age mean ± SD according to disease grade (1, 2 and 3): mean ± SD (n): 36.8 ± 11.2 (5), 48.0 ± 19.6 (5) and 46.5 ± 11.0 (6), respectively; *p*-value = 0.422, or disease stage (I/II and III/IV): mean ± SD (n): 42.5 ± 16.4 (9) and 45.7 ± 11.7 (7), respectively; *p*-value = 0.675.

**Table 1:** Clinicopathological parameters of patient groups

	Patient group		*p*-value
	Benign	Malignant	
	(*N* = 14)	(*N* = 16)	
Age (mean ± SD)	45.2 ± 12.7	43.9 ± 14.2	0.788^c^
Menstrual cycle			
Premenopausal	7	11	0.296^a^
Postmenopausal	7	5	
Ascites			
Absent	13	8	0.011^a^
Present	1	8	
Serum marker (median)			
CA125 (U/ml)	11.6	186.0	0.001^b^
KLK10 (ng/ml)	1.41	1.99	0.236^b^
KLK6 (ng/ml)	3.05	3.17	0.678^b^

*SD* standard deviation

^a^χ^*2*^ test

^b^Mann-Whitney test

^**c**^Student *t*-test

Each patient participated by one tumor sample. In addition, samples from contralateral normal ovaries of some patients in benign and malignant patient groups were included as normal controls (*n* = 7 and 6, respectively). Table 2 shows the pathological characteristics of tumor samples in the two patient groups. Histopathological H&E examination of all samples showed good quality of the tissue samples and confirmed the previously known diagnoses.

**Table 2:** Pathological characteristics of tumor samples

		Benign tumors	Malignant tumors
		(*N*, %)	(*N*, %)
Pathology subgroup/subtype		Neoplastic (14, 100%)	Epithelial (11, 68.75%)
		Serous cystadenoma (3)	Serous (4)
		Serous cystadenofibroma (1)	Mucinous (2)
		Simple serous cyst (2)	Endometrioid (4)
		Granulosa cell tumor (3)	Clear cell (1)
		Mature cystic teratoma (3)	Non-epithelial (3, 18.75%)
		Mucinous cystadenoma (1)	Yolk sac tumor (1)
		Brenner tumor (1)	Liposarcoma (1)
			Sretoli-leydig cell tumor (1)
			Metastatic (2, 12.5%)
			Colon cancer (2)
Grade			
	1	–	5 (31.3%)
	2	–	5 (31.3%)
	3	–	6 (37.5%)
Stage			
	I/II	–	9 (56.3%)
	III/IV	–	7 (43.8%)

### MSP

In the overall there were 42 unmethylated-specific positive PCR tests and only one methylated-specific positive PCR test. The unmethylated PCR band size was as follows: min.-max.: 101.30–274.00 bp; median: 123.45 bp and mean ± SD: 126.25 ± 23.61 bp, whereas the methylated band was 134.5 bp. The methylation positive control sample was positive for the methylated-specific PCR (band size: 129.0 bp) but negative for unmethylated-specific PCR.

**Fig. 1:**
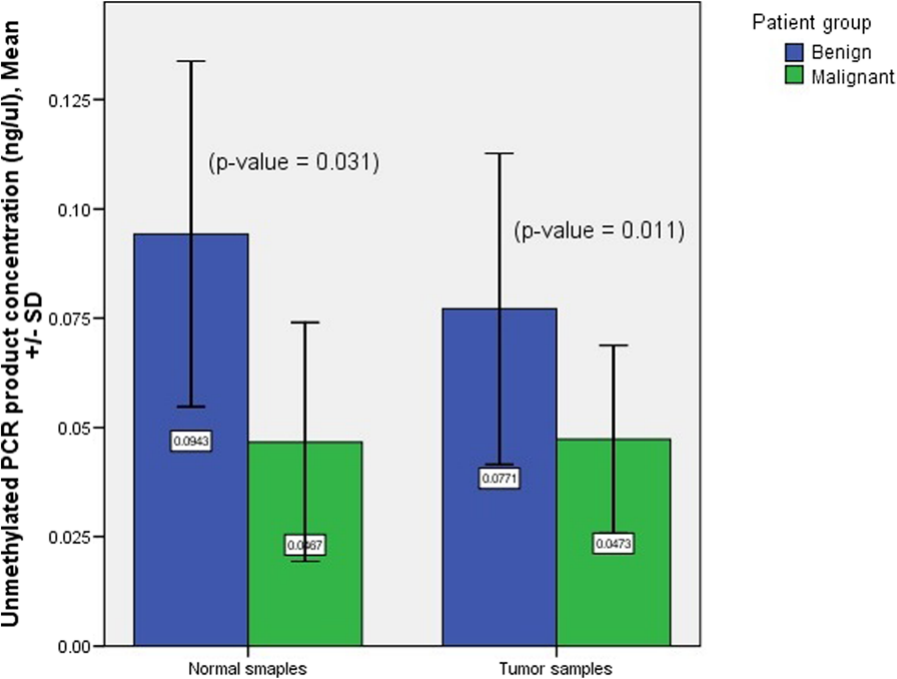
*KLK10* exon 3 unmethylated PCR product concentration (ng/μl) mean ± SD in patient group samples. Number of samples in benign and malignant patient groups were: normal sample: 7 and 6, respectively, and tumor sample: 14 and 15, respectively

Qualitative results of MSP did not show statistically significant difference in the two patient groups for both tumor and normal samples (Table 3). However, comparison of *KLK10* exon 3 unmethylated PCR product concentration mean ± SD (ng/μl) showed statistically significant differences in benign (*n* = 14), malignant (*n* = 15) and overall normal (*n* = 13) samples; mean ± SD: 0.077 ± 0.035, 0.047 ± 0.021, and 0.072 ± 0.041, respectively; *p*-value = 0.046. Moreover, *KLK10* exon 3 unmethylated PCR product concentration mean ± SD (ng/μl) showed statistically significant differences in tumor and normal samples in the two patient groups (Fig. 1).

**Table 3:** MSP qualitative results in patient group samples

MSP	Normal samples		Tumor samples	
	Benign patient	Malignant patient	Benign patient	Malignant patient
Methylated-specific PCR				
Positive	0	0	0	1^b^
Negative	7	6	14	15
*p*-value	–		0.341^a^	
Unmethylated-specific PCR				
Positive	7	6	14	15
Negative	0	0	0	1^b^
*p*-value	–		0.341^a^	

- not applicable

^a^χ^*2*^ test

^b^The two odd results in the malignant samples belonged to two samples; one was doubly positive (ovarian serous adenocarcinoma) and the other was doubly negative (Sertoli-Leydig cell tumor) for the two MSP test types

Spearman’s correlation of *KLK10* exon 3 unmethylated PCR product concentration (ng/μl) showed statistically significant positive correlation with patient age (ρ = 0.398; *p*-value = 0.009) and statistically significant negative correlation with serum KLK10 (ρ = − 0.300; *p*-value = 0.054) but not KLK6 (ρ = − 0.114; *p*-value = 0.471) or CA125 (ρ = − 0.214; p-value = 0.197). Comparisons of *KLK10* exon 3 unmethylated PCR product concentration mean ± SD (ng/μl) in overall samples and in sample pathology types in the two patient groups according to patient age and serum KLK10 are shown in Tables 4 and 5, respectively.

**Table 4:** Comparison of *KLK10* exon 3 unmethylated PCR product concentration (ng/μl) according to patient age

*KLK10* exon 3 unmethylated PCR product; ng/μl, mean ± SD	Age group			*p*-value
	16–32 years	33–55 years	> 55 years	
Overall samples (*n* = 6–27)	0.032 ± 0.029	0.064 ± 0.029	0.090 ± 0.037	0.004^b^
Benign patients:				
Normal ovary (*n* = 0–5)	–	0.078 ± 0.034	0.135 ± 0.007	0.078^b^
Benign ovary (*n* = 2–10)	0.060 ± 0.042	0.078 ± 0.034	0.090 ± 0.056	0.728^b^
*p*-value	–	1.00^a^	0.380^a^	
Malignant patients:				
Normal ovary (*n* = 1–3)	0.020 ± −	0.040 ± 0.000	0.070 ± 0.042	0.335^b^
Malignant ovary (*n* = 3–9)	0.017 ± 0.011	0.049 ± 0.010	0.073 ± 0.015	0.000^b^
*p*-value	0.826^a^	0.188^a^	0.903^a^	

- no samples or statistical test not applicable, *SD* standard deviation

^a^Student *t*-test

^b^ANOVA test

**Table 5:** Comparison of *KLK10* exon 3 unmethylated PCR product concentration (ng/μl) according to preoperative serum KLK10

*KLK10* exon 3 unmethylated PCR product; ng/μl, mean ± SD	Serum KLK10		*p*-value
	< 2 ng/ml	≥ 2 ng/ml	
Overall samples (*n* = 15–27)	0.075 ± 0.038	0.046 ± 0.018	0.007^a^
Benign patients:			
Normal ovary (*n* = 1–6)	0.100 ± 0.040	0.060 ± −	0.397^a^
Benign ovary (*n* = 3–11)	0.086 ± 0.033	0.043 ± 0.023	0.060^a^
*p*-value	0.463^a^	0.596^a^	
Malignant patients:			
Normal ovary (*n* = 2–4)	0.070 ± 0.042	0.035 ± 0.010	0.153^a^
Malignant ovary (*n* = 7–8)	0.044 ± 0.023	0.051 ± 0.019	0.511^a^
*p*-value	0.252^a^	0.156^a^	

- not applicable, *SD* standard deviation

^a^Student *t*-test

### ROC curves

**Fig. 2:**
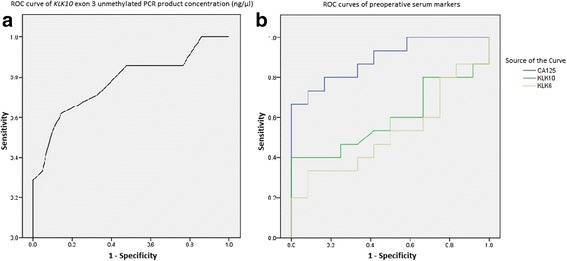
ROC curve analysis of *KLK10* exon 3 unmethylated PCR product concentration (ng/μl) in overall patient group samples (**a**) and ROC curves of preoperative serum markers CA125, KLK10 and KLK6 in patient groups (**b**). Markers’ diagnostic profiles were as follows:

ROC curves for *KLK10* exon 3 unmethylated PCR product concentration (ng/μl) and preoperative serum markers in the two patient groups are shown in Figs. 2a and b, respectively.

Combination of *KLK10* exon 3 unmethylated PCR product concentration and serum marker showed enhanced combined marker sensitivity with all 3 markers and better specificity with CA125 but not KLK10 or KLK6. Combined marker profile of *KLK10* exon 3 unmethylated PCR product concentration with serum marker was as follows; CA125: sensitivity: 100%, specificity: 94.4%, PPV: 68.9%, NPV: 73.9% and accuracy: 71.1%; KLK10: sensitivity: 90.4%, specificity: 85.7%, PPV: 67.8%, NPV: 62% and accuracy: 64.9%; and KLK6: sensitivity: 85.7%, specificity: 71.4%, PPV: 60%, NPV: 60% and accuracy: 60%.

### Patients’ survival

Five-year patient follow-up data were available between 2007 and 2012. Five patients died within the first two years of the follow-up period. In the living 11 patients there were 9 disease-free and 2 relapsed. Comparisons of patient clinicopathological parameters according to patient survival (living and died) showed statistically significant differences in preoperative serum CA125 concentration (U/ml); median (n): 101.25 (10) and 1252 (5), respectively; *p*-value = 0.037, and disease stage (I/II and III/IV): late stage was more frequent in died patients compared to the living (4 vs. 1, respectively); *p*-value = 0.049. The *p*-values for other clinicopathological parameters were as follows: patient age mean ± SD: 0.355; serum KLK10 (ng/ml) mean ± SD: 0.798; serum KLK6 (ng/ml) mean ± SD: 0.278; ascites (absent and present): 0.106; disease grade (1, 2, and 3): 0.622; pathology subgroup (epithelial, nonepithelial and metastatic): 0.402; and epithelial pathology subtype (serous, mucinous, endometrioid and clear cell carcinoma): 0.735.

Comparison of *KLK10* exon 3 unmethylated PCR product concentration (ng/μl) mean ± SD according to patient survival (living and died) did not show statistically significant differences in overall malignant patient samples: mean ± SD (n): 0.042 ± 0.015 (14) and 0.055 ± 0.032 (7), respectively; *p*-value = 0.228, normal samples: mean ± SD (n): 0.035 ± 0.010 (4) and 0.070 ± 0.042 (2), respectively; *p*-value = 0.153, or malignant samples: mean ± SD (n): 0.046 ± 0.017 (10) and 0.050 ± 0.030 (5), respectively; *p* = 0.748. However, the oddly methylated-specific PCR positive sample in our results was serous carcinoma of grade 3, stage III/IV and its unmethylated PCR product concentration was 0.06 ng/μl; the patient of which died one year after diagnosis and start of treatment.

## Discussion

In the literature we could find only one study on *KLK10* exon 3 methyaltion in primary ovarian tumors that was reported by Sidiropoulos and colleagues [25]. Our results agree with those in the previous work with some logic approximation due to differences in the used technique for gene methylation assessment (MSP vs. direct DNA sequencing, respectively) and the examined *KLK10* expression parameter (serum KLK10 vs. cytosolic KLK10, respectively). The two studies showed *KLK10* exon 3 methylation only in malignant samples (methylated-specific PCR positive samples: 1/16 and samples with partially methylated *KLK10* exon 3: 6/7, respectively) but not in normal samples (methylated-specific PCR negative samples: 13/13 and samples with fully unmethylated *KLK10* exon 3: 2/2, respectively). Additionally, we studied *KLK10* exon 3 methylation in benign ovarian tumors – not included in the previous work – and they all were unmethylated (Table 3). Differences in the internal working of MSP and direct DNA sequencing would make comparison of frequencies of methylated samples in the two studies practically unfeasible (see later).

Moreover, as for the correlation between *KLK10* gene expression and *KLK10* exon 3 methylation the two studies showed similar results. In the previous work there was fair *KLK10* expression in normal samples (fully unmethylated) and high expression in the partially methylated malignant samples (3 out of 6). In concordance to those results our samples showed negative correlation between serum KLK10 level (ng/ml) and *KLK10* exon 3 unmethylated PCR product concentration (ng/μl) by Spearman’s correlation and by comparing the unmethylated PCR product concentration mean ± SD in overall samples according to KLK10 level (Table 5).

While our qualitative MSP results did not show statistically significant differences in patient group samples (Table 3), *KLK10* exon 3 unmethylated PCR product concentration (ng/μl) mean ± SD showed statistically significant differences in normal and tumor samples in the two patient groups (Fig. 1). Because our MSP experiment conditions were almost constant for all PCR tests (including the operator, instruments and machines, kits and reagents, DNA template concentration, PCR program, simultaneous unmethylated- and methylated-specific PCRs, and random sample sets in 4 PCR runs done in 4 different days) those statistically significant differences of *KLK10* exon 3 unmethylated PCR product concentration should be due to differences in the methylation patterns of samples rather than due to unfounded chance (see later).

Individual cells in the same tissue may show heterogeneity as regard their DNA methylation patterns [28]. Indeed, understanding that methylation pattern heterogeneity is fundamental in interpreting qualitative and quantitative DNA methyaltion data and also for appreciating and understanding several aspects of gene functions. Each cell contains two copies of each CpG site; one on each of the two homologous chromosomes, and, therefore, can have on its own 3 different methylation patterns for that particular CpG site: fully unmethylated, fully methylated or partially methylated. The possible methylation patterns of a number (n) of CpG sites are given mathematically as 2^n^. For example, when studying an average number of 12 CpGs by MSP, the possible methylation patterns in the DNA sample are 2^12^ = 4096, which reflects possible immense methylation pattern heterogeneity when the cell can have any two of these patterns. However, in the context of *KLK10* exon 3 in ovarian cancer the property of methylation pattern heterogeneity would be present almost exclusively in malignant samples but not in normal ones because in the previous work the malignant samples were partially methylated, whereas the normal samples were fully unmethylated [25].

**Fig. 3:**
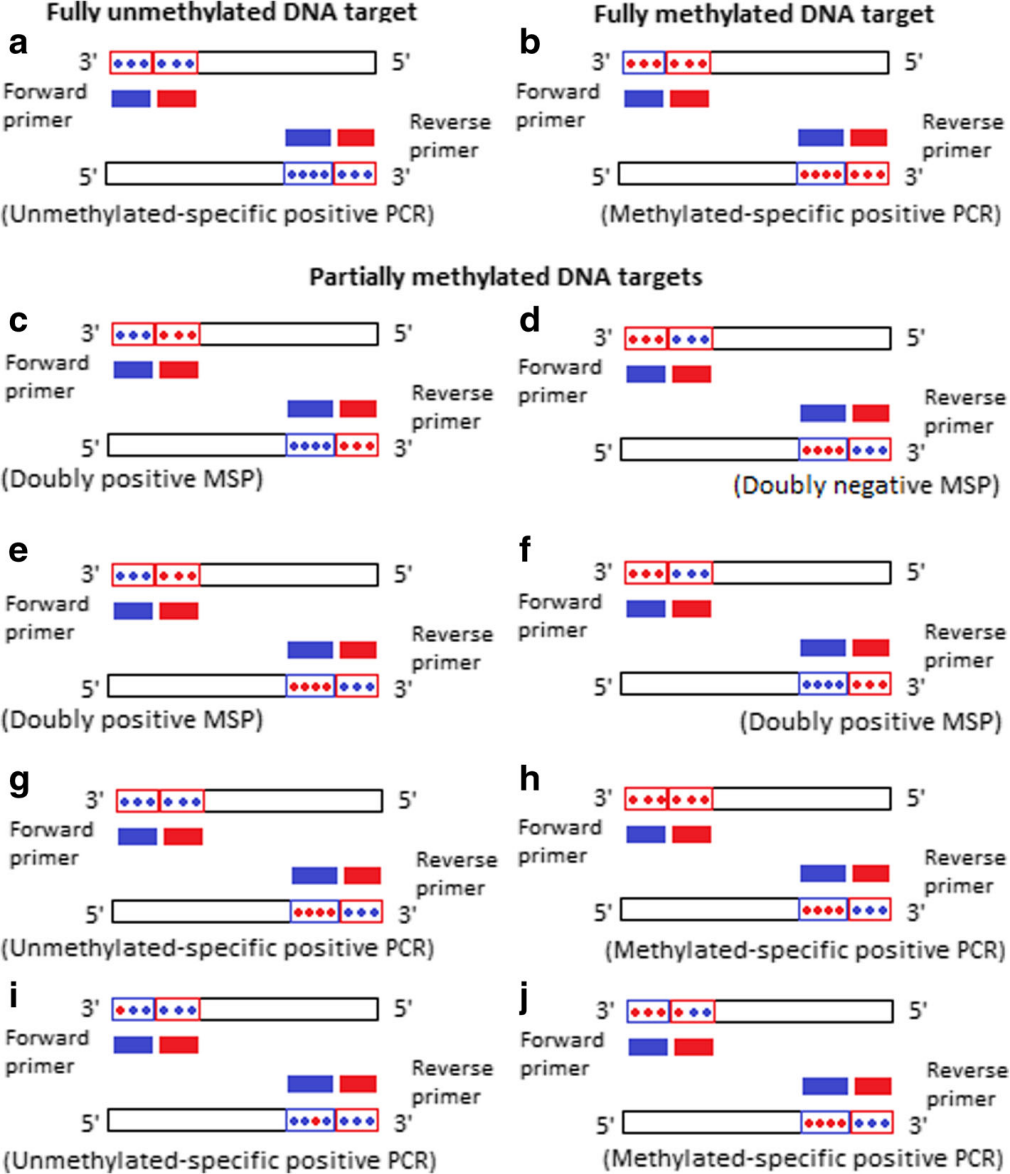
The Figure shows a schematic representation of a theoretical rationale for MSP results based on previous works [23, 28, 29]. The DNA templates (long bars with 3′- > 5′ directionality designation) show blue and red dots representing unmethylated and methylated CpGs, respectively. The short blue and red bars represent unmethylated- and methylated-specific primer pairs, respectively. For simplicity, we show methylation patterns with uniform methylation status at the primer’s annealing site on DNA template (**a**-**h**) and only two with non-uniform methylation status (**i** and **j**). While fully unmethylated (**a**) and fully methylated (**b**) patterns provide unmethylated- and methylated-specific positive PCRs, respectively, partially methylated patterns (**c**-**j**) can have any of the four possible qualitative MSP results. MSP-inexpressible partially methylated pattern (**d**) may rationalize our doubly negative malignant sample (Sertoli-Leydig cell tumor), in addition to possible mutation as methylated CpGs are known hotspot for mutations [39, 40]

In Fig. 3 we present a theoretical rationale for qualitative and quantitative MSP results based on possible methylation pattern heterogeneity in individual cells of a sample [28], the independency of the primer pairs in MSP from one another [23, 29] and previous DNA methylation results by MSP and direct DNA sequencing in cell lines and primary breast cancer [23]. We hypothesize that the existence of different partially methylated patterns in the sample would result in the stratification of the starting DNA template into template classes with varying MSP-expressivities according to the number of methylation-wise matching CpG sites with their corresponding nucleotides in the respective PCR primer. While some patterns would be more or less favorable for primer annealing (Fig. 3: elements C, and E-J), other patterns may not be at all (Fig. 3: element D). Differences of the proportions of those differentially MSP-expressible partially methylated patterns in the samples may be effected practically as differences in the unmethylated PCR product concentration. Given that those partially methylated patterns would occur only in malignant samples but not in normal – and probably also benign – samples (see above), such proportionate decreases in the unmethylated PCR product concentration would happen in malignant samples only.

Our results showed statistically significant positive correlation between *KLK10* exon 3 unmethylated PCR product concentration (ng/μl) and patient age by Spearman’s correlation and on comparing its mean ± SD in overall samples and in malignant samples according to age groups (Table 4). However, because there was no statistically significant difference of patient age in the two patient groups (Table 1), comparison of *KLK10* exon 3 unmethylated PCR product concentration in patient group samples should be valid and justifiable.

*KLK10* exon 3 methylation correlated to high disease grade (breast cancer [30]), late stage (lung cancer [26]) and bad patient prognosis (acute lymphoblastic leukemia [24]). Similarly, the only methylated-specific PCR positive sample in our results was serous carcinoma of high grade (3) and late stage (III/IV) whose patient died within one year after starting the treatment. However, in our results comparison of *KLK10* exon 3 unmethylated PCR product concentration (ng/μl) according to disease grade, stage or patient survival did not show statistically significant differences that may be attributable to early implication of deregulated *KLK10* exon 3 methylation in the disease or lack of significant information about site-specific CpG methylation in MSP data (see later).

The notion that information about the exact location of DNA methylation maybe crucial for correct clues about disease diagnosis, prognosis and gene function [31] would greatly restrain the translational impact of DNA methylation data by MSP. For example, lack of site-specific CpG methylation data (in MSP) and/or data about their pattern distribution in the sample (in MSP and direct DNA sequencing, respectively) may explain – at least in part – the inconsistent results, in sample subsets or sample types, about the downregulation of *KLK10* through *KLK10* exon 3 hypermethylation in previous works [23–26]. At the functional aspect, the reported antitumor role of *KLK10* was demonstrated in vector-mediated transfection experiments in vitro and/or in tumor xenograft in mice using different cancer cell lines; e.g. breast [32], prostate [16] and gastric cell [33], including ovarian cell line [34]. Noteworthy is that overexpression of *KLK10* was found in malignant sample subsets (tissues or sera) in cancer types in which *KLK10* was shown experimentally as tumor suppressor; breast [35], prostate [19], gastric [36] and ovary [20, 27]. Taken together, the antitumor role of *KLK10* may be understood only in relation to the context of the given study as regard type of tissue and the underlying pathophysiologic processes [37].

Indeed, the translational impact of DNA methylation data can vary greatly with the pros and cons of the used tools. While MSP studies only a few of CpGs without regard to their exact location, direct DNA sequencing can give site-specific CpG methylation data for all CpGs of interest as percent methylation in the sample. Unfortunately, both tools fall short of providing information about site-specific CpG methylation pattern distribution in sample. The more recently discovered next generation sequencing have the advantage of presenting site-specific DNA methylation data in absolute numbers and may tackle the problem of methylation pattern distribution in hand of sophisticated computational methods [38]. Nonetheless, considering possible dynamicity of DNA methylation as a mechanism for gene regulation [1] another dimension for DNA methylation pattern resolution may be necessary in order to spot and trace such spatiotemporal DNA methylation events rather than making statements based merely on single static snapshot image.

## Conclusions

In summary, our results agreed with those in the previous work as for the exclusive occurrence of *KLK10* exon 3 methylation in malignant but not in normal ovarian samples. Additionally, we assessed *KLK10* exon 3 methylation in benign ovarian tumors (not included in the previous work) and found that they were unmethylated. Although our qualitative MSP results did not show statistically significant differences in patient group samples, semi-quantitative *KLK10* exon 3 unmethylated PCR product concentration (ng/μl) showed statistically significant differences not only in tumor samples but also in otherwise histologically normal samples in benign and malignant patients. These findings would suggest, for the first time, *KLK10* exon 3 unmethylated PCR product concentration as potential early epigenetic diagnostic marker in ovarian cancer. The diagnostic potential of *KLK10* exon 3 unmethylated PCR product concentration in overall patient group samples was also evident in ROC curve analysis, and it could enhance the sensitivity of preoperative serum markers CA125, KLK10 and KLK6. Taking into account the limitations in the present study (small sample size and semi-quantitative assessment of PCR product concentration) further studies are needed before making a statement about the validity and reproducibility of these results. Moreover, we highlighted the translational limitations of DNA methylation data in MSP and direct DNA sequencing and addressed future challenges of the more recent next generation sequencing (NGS) to further enhance the translational impact of DNA methylation data.

## Abbreviations

5-MC: 5-methylcytosine
bp: Base pair
CA125: Cancer antigen 125
DNA: Deoxyribonucleic acid
DNMT1: DNA methyltransferase 1
DNMT3a: DNA methyltransferase 3a
DNMT3b: DNA methyltransferase 3b
FFPE: Formalin-fixed paraffin-embedded
H&E: Hematoxylin and eosin
KLK10: Kallikrein-related peptidase 10
*KLK10*: Kallikrein-related peptidase10 gene
KLK6: Kallikrein-related peptidase 6
MECs: Human mammary epithelial cells
micRNA: micro RNA
MSP: Methylation-specific PCR
NEC: Human normal epithelial cell line
NGS: Next generation sequencing
NPV: Negative predictive value
PCR: Polymerase chain reaction
PPV: Positive predictive value
SAM: S-adenosylmethionine
